# Supplementary material for: Defining the multifaceted roles of dental academics: A consensus approach using the Fuzzy Delphi Method
Source: PLoS One. 2025 Aug 22;20(8):e0330636. doi: 10.1371/journal.pone.0330636 (PMC12373288; doi:10.1371/journal.pone.0330636)
Supplement: Attachment — Submitted filename: PONE-D-24-57785_reviewer.pdf [file pone.0330636.s002.pdf]

PLOS ONE

Defining the Multifaceted Roles of Dental Academics: A Consensus Approach Using the Fuzzy Delphi Method

--Manuscript Draft--

Manuscript Number:	PONE-D-24-57785
Article Type:	Research Article
Full Title:	Defining the Multifaceted Roles of Dental Academics: A Consensus Approach Using the Fuzzy Delphi Method
Short Title:	Defining dental academic roles through Fuzzy Delphi Method
Corresponding Author:	Norasmatul Akma Ahmad, MClinDent<br>Universiti Malaya Fakulti Pergigian<br>kuala Lumpur, MALAYSIA
Keywords:	fuzzy delphi method<br>dental academics<br>definition roles<br>academician<br>educator clinician<br>administrator<br>dental educator<br>clinical<br>academics

Powered by Editorial Manager® and ProduXion Manager® from Aries Systems Corporation

Abstract:	Dental academics are the foundation of dental institutions. They are mostly dental specialists and scientists driven by a passion for teaching and intellectual challenges. Although most have not been trained or certified as educators, they are tasked with educating the next generation of dentists within their fields of expertise. Beyond teaching, dental academics juggle multifaceted roles as educators, researchers, administrators, and clinicians. The strive to balance these multiple roles may lead dental academics to face identity conflicts, loss of work satisfaction and ultimately burnout and attrition from the workforce. Given the limited literature on defining faculty competencies and roles in medical and dental education, and an ongoing retention issues, it is crucial to clearly delineate these roles to assist dental academics establish their academic identity and plan their careers. This study aims to systematically define the specific roles of dental academics using the Fuzzy Delphi Method (FDM). Engaging 27 experts in the first round and 23 in the second, consensus was reached on items derived from evidence-based literature to define these roles. Analysis yielded 17 accepted items representing the four main roles of dental academics. Each primary role encompassed a diverse range of job descriptions, precluding a single, definitive description. The application of FDM not only refines the understanding of academic roles but also contributes to establishing the identity of dental academics, aiding their adaptation to the multiple roles and supporting their career advancement.<br><br>Enable GingerCannot connect to Ginger Check your internet connection

	or reload the browserDisable in this text fieldEditEdit in GingerEdit in Ginger×
Order of Authors:	Norasmatul Akma Ahmad, MClinDent, MRDRCS
	Noor Hayaty Abu Kasim, PhD
	Sabri Musa, PhD
	Siti Adibah Othman, DDS, MOrthRCS
	Zahra Naimie, PhD
Additional Information:	
Question	Response

Powered by Editorial Manager® and ProduXion Manager® from Aries Systems Corporation

Financial Disclosure<br><br>Enter a financial disclosure statement that describes the sources of funding for the work included in this submission. Review the submission guidelines for detailed requirements. View published research articles from PLOS ONE for specific examples.<br><br>This statement is required for submission and will appear in the published article if the submission is accepted. Please make sure it is accurate.<br><br>Funded studies<br>Enter a statement with the following details:<br>• Initials of the authors who received each award<br>• Grant numbers awarded to each author<br>• The full name of each funder<br>• URL of each funder website<br>• Did the sponsors or funders play any role in the study design, data collection and analysis, decision to publish, or preparation of the manuscript?<br><br>Did you receive funding for this work?	The author(s) received no specific funding for this work.
Competing Interests<br><br>Use the instructions below to enter a competing interest statement for this submission. On behalf of all authors, disclose any competing interests that could be perceived to bias this work—acknowledging all financial support and any other relevant financial or nonfinancial competing interests.	The authors have declared that no competing interests exist.

Powered by Editorial Manager® and ProduXion Manager® from Aries Systems Corporation

This statement is required for submission and will appear in the published article if the submission is accepted. Please make sure it is accurate and that any funding sources listed in your Funding Information later in the submission form are also declared in your Financial Disclosure statement.

View published research articles from PLOS ONE for specific examples.

NO authors have competing interests

Enter: The authors have declared that no competing interests exist.

Authors with competing interests

Enter competing interest details beginning with this statement:

I have read the journal's policy and the authors of this manuscript have the following competing interests: [insert competing interests here]

* typeset

Powered by Editorial Manager® and ProduXion Manager® from Aries Systems Corporation

Ethics Statement<br><br>Enter an ethics statement for this submission. This statement is required if the study involved:<br><br>• Human participants<br>• Human specimens or tissue<br>• Vertebrate animals or cephalopods<br>• Vertebrate embryos or tissues<br>• Field research<br><br>Write "N/A" if the submission does not require an ethics statement.<br><br>General guidance is provided below. Consult the submission guidelines for detailed instructions. Make sure that all	Ethical approval for this study was granted by the Medical Ethics Committee, Faculty of Dentistry, Universiti Malaya (Ethics No: DF RD 2212/0070 [L]). Data collection was conducted from 31st January 2023 to 1st April 2023, with all participants providing informed consent through an online platform.

Powered by Editorial Manager® and ProduXion Manager® from Aries Systems Corporation

information entered here is included in the Methods section of the manuscript.<br><br>Format for specific study types<br><br>Human Subject Research (involving human participants and/or tissue)<br>• Give the name of the institutional review board or ethics committee that approved the study<br>• Include the approval number and/or a statement indicating approval of this research<br>• Indicate the form of consent obtained (written/oral) or the reason that consent was not obtained (e.g. the data were analyzed anonymously)<br><br>Animal Research (involving vertebrate animals, embryos or tissues)<br>• Provide the name of the Institutional Animal Care and Use Committee (IACUC) or other relevant ethics board that reviewed the study protocol, and indicate whether they approved this research or granted a formal waiver of ethical approval<br>• Include an approval number if one was obtained<br>• If the study involved non-human primates, add additional details about animal welfare and steps taken to ameliorate suffering<br>• If anesthesia, euthanasia, or any kind of animal sacrifice is part of the study, include briefly which substances and/or methods were applied<br><br>Field Research<br><br>Include the following details if this study involves the collection of plant, animal, or other materials from a natural setting:<br>• Field permit number<br>• Name of the institution or relevant body that granted permission	
Data Availability<br><br>Authors are required to make all data underlying the findings described fully available, without restriction, and from the time of publication. PLOS allows rare exceptions to address legal and ethical	Yes - all data are fully available without restriction

Powered by Editorial Manager® and ProduXion Manager® from Aries Systems Corporation

concerns. See the PLOS Data Policy and FAQ for detailed information.<br><br>A Data Availability Statement describing where the data can be found is required at submission. Your answers to this question constitute the Data Availability Statement and will be published in the article, if accepted.<br><br>Important: Stating ‘data available on request from the author’ is not sufficient. If your data are only available upon request, select ‘No’ for the first question and explain your exceptional situation in the text box.<br><br>Do the authors confirm that all data underlying the findings described in their manuscript are fully available without restriction?	

Powered by Editorial Manager® and ProduXion Manager® from Aries Systems Corporation

Describe where the data may be found in full sentences. If you are copying our sample text, replace any instances of XXX with the appropriate details.<br><br>• If the data are held or will be held in a public repository, include URLs, accession numbers or DOIs. If this information will only be available after acceptance, indicate this by ticking the box below. For example: All XXX files are available from the XXX database (accession number(s) XXX, XXX.).<br>• If the data are all contained within the manuscript and/or Supporting Information files, enter the following: All relevant data are within the manuscript and its Supporting Information files.<br>• If neither of these applies but you are able to provide details of access elsewhere, with or without limitations, please do so. For example:<br><br>Data cannot be shared publicly because of [XXX]. Data are available from the XXX Institutional Data Access / Ethics Committee (contact via XXX) for researchers who meet the criteria for access to confidential data.	All relevant data are within the manuscript and its Supporting Information files.

The data underlying the results presented in the study are available from (include the name of the third party and contact information or URL).<br>• This text is appropriate if the data are owned by a third party and authors do not have permission to share the data.<br><br>* typeset	
Additional data availability information:	

Powered by Editorial Manager® and ProduXion Manager® from Aries Systems Corporation

Manuscript

Click here to access/download;Manuscript;23.12.24-Defining the Multifaceted Roles of Dental Academics.pdf

Defining the Multifaceted Roles of Dental Academics: A Consensus Approach Using the Fuzzy Delphi Method

Norasmatul Akma Ahmad [1*], Noor Hayaty Abu Kasim [1*], Sabri Musa [2*] , Siti Adibah Othman [2*], Zahra Naimie [3*]

1. Department of Restorative Dentistry
Faculty of Dentistry
Universiti Malaya
Kuala Lumpur , MY 50603

2. Department of Paediatric Dentistry and Orthodontics,
Faculty of Dentistry
Universiti Malaya
Kuala Lumpur, MY 50603
3. Universiti Malaya
Kuala Lumpur, MY 50603

*Corresponding authors :

Corresponding email: akma@um.edu.my (NAA)
Corresponding email: nhayaty@um.edu.my (NHAK)
Corresponding email: sabrim@um.edu.my (SM)
Corresponding email: sitiadibah@um.edu.my (SAO) Corresponding email: znaimie@gmail.com (ZN)

Declaration of Interest:

The authors report no conflicts of interest. The authors alone are responsible for the content and writing of the paper.

Defining the Multifaceted Roles of Dental Academics: A Consensus Approach Using the Fuzzy Delphi Method

Abstract:

Dental academics are the foundation of dental institutions. They are mostly dental specialists and scientists driven by a passion for teaching and intellectual challenges. Although most have not been trained or certified as educators, they are tasked with educating the next generation of dentists within their fields of expertise. Beyond teaching, dental academics juggle multifaceted roles as educators, researchers, administrators, and clinicians. The strive to balance these multiple roles may lead dental academics to face identity conflicts, loss of work satisfaction and ultimately burnout and attrition from the workforce. Given the limited literature on defining faculty competencies and roles in medical and dental education, and an ongoing retention issues, it is crucial to clearly delineate these roles to assist dental academics establish their academic identity and plan their careers. This study aims to systematically define the specific roles of dental academics using the Fuzzy Delphi Method (FDM). Engaging 27 experts in the first round and 23 in the second, consensus was reached on items derived from evidencebased literature to define these roles. Analysis yielded 17 accepted items representing the four main roles of dental academics. Each primary role encompassed a diverse range of job descriptions, precluding a single, definitive description. The application of FDM not only refines the understanding of academic roles but also contributes to establishing the identity of dental academics, aiding their adaptation to the multiple roles and supporting their career advancement.

Introduction

Dental academics play a pivotal role in shaping the future of dental education, serving as the foundation of educational institutions while navigating a complex array of responsibilities that extend well beyond traditional teaching. These professionals, often specialists in their respective fields, are charged with the critical task of preparing the next generation of dentists, despite many lacking formal pedagogical training [1]. The assumption that clinical expertise inherently qualifies them as educators can lead to a reliance on generic teaching principles, which may not adequately address the unique challenges of dental education [2,3], highlighting the need for professional development to enhance their effectiveness as teachers [4]. However, teaching is merely one facet of their multifaceted roles. In addition to instruction, dental academics are frequently involved in research, administrative duties, and clinical practice [5,6], which necessitates targeted professional development and institutional support.

Commented [sm1]: Considering that the focus of the study is on defining the roles. What are the views regarding these roles.
A brief elaboration on them as was done above for teaching.

Despite the growing recognition of the importance of faculty development programs tailored to the diverse skill sets required by dental educators [7], literature addressing the definition of faculty roles in dental education remains scarce. Scott (2003) identified four primary roles namely, teaching, research, clinical practice, and administration [8], while Hands (2006) proposed a tripartite classification encompassing clinical teachers, clinical scholars, and research-intensive scholars [9]. More recent classifications have reiterated these roles but emphasized the expectation for dental academics to balance multiple responsibilities due to their interconnected nature [5]. The struggle to manage diverse roles can lead to professional ambiguity, diminished job satisfaction, and ultimately contribute to faculty burnout and attrition within the dental workforce [10,11,12].

Commented [sm2]: What are these multiple responsibilities that academics are balancing?

Commented [sm3]: Is it the struggle to manage the diverse roles or is that the roles are not well defined? You can elaborate on how managing diverse roles may lead to professional ambiguity?

A clearer understanding and definition of dental academic roles are essential not only for enhancing faculty satisfaction and productivity but also for fostering career progression among dental academics. By delineating and reinforcing the distinct responsibilities associated with teaching, research, administration and clinical practices, institutions can provide targeted professional development opportunities that support faculty retention and facilitates the

establishment of faculty's academic and professional identities. Academic identity encompasses self-definition and self-understanding shaped by social institutions and individual relationships [10] while professional identity is rooted in vocational self-concept and is regarded as an individual's role within a workplace [13].

A nuanced comprehension of academic roles, attributes, and competencies can guide dental academics in solidifying their academic and professional identities. Encouraging individuals to cultivate their skills across various roles will empower them to navigate their academic careers effectively [14]. Moreover, while identity is not the sole determinant of career success, it significantly influences motivation for learning and professional development goals [15], ultimately impacting recognition and rewards within academia [16]. A clear definition of dental academics' roles can foster a well-defined academic identity, which in turn enhances self efficacy and job motivation, ultimately contributing to greater career success [17,18].

Commented [sm4]: This seem to be contradicting the statement highlighted in yellow above.

This study aims to systematically define the specific roles of dental academics as part of a broader investigation into their attributes, competencies, and career development. By establishing a comprehensive framework for understanding these multifaceted roles, we anticipate creating a robust self-reflective instrument that will aid dental academics in navigating their professional journeys.

Commented [sm5]: Was this achieved? I did not see this reflected anywhere? And also if yes how is the instrument self-reflective?

Materials and methods

Ethical approval

Ethical approval for this study was granted by the Medical Ethics Committee, Faculty of Dentistry, Universiti Malaya (Ethics No: DF RD 2212/0070 [L]). Data collection was conducted from 31st January 2023 to 1st April 2023, with all participants providing informed consent through an online platform.

This study employed the Fuzzy Delphi Method (FDM), an advanced version of the traditional Delphi method that integrates fuzzy set theory to address the ambiguities often present in expert opinions [19]. The FDM involves a series of stages that must be followed for a study to be considered empirical, with seven specific steps outlined in FDM [20] (see Table 1).

Table 1: The FDM Steps

STEPS	PROCEDURES
Step 1	Preparation of Fuzzy Delphi questionnaires either through literature review, expert interviews, or focus groups discussion.
Step 2	Identification of experts and the number of experts who will be appointed to assess the importance of evaluation criteria for elements, items, issues, variables, and other variables that will be measured using linguistic variables needs to be determined.
Step 3	All linguistic variables are converted to triangular fuzzy numbers.
Step 4	Conversion of the Likert scale data into a fuzzy scale and then, the threshold is calculated, i.e., (d) value based on this formula: $$d(\tilde{m},\tilde{n}) = \sqrt{\frac{1}{3}[(m_1 - n_1)^2 + (m_2 - n_2)^2 + (m_3 - n_3)^2]}$$ This process involves converting all scales of linguistic variables to Fuzzy triangle numbering (Triangular Fuzzy Numbers). The values of m_1, m_2 and m_3 are used to represent the Triangular Fuzzy Number. The value of m_1 relates to the minimum value, m_2 to the most appropriate value, and m_3 to the maximum value
Step 5	Determination of the first condition which is when the difference between the average and expert evaluation data is less than or equal to the threshold value, (d) = 0.2, all experts are said to have achieved an agreement.
Step 6	Determination of the second condition, which is the percentage value for expert agreement must be more than or equal to 75.0%.
Step 7	Defuzzification process which analyses data using an average of fuzzy numbers or an average response to identify the value of the fuzzy score and the rank and priority of each item, element, issue, and so on calculated based on this formula: $$\mathrm{A} = \frac{1}{3}(\mathrm{m}_1 + \mathrm{m}_2 + \mathrm{m}_3)$$

Commented [sm6]: What determined the threshold value to be 0.2

Fuzzy Delphi Method (FDM) Questionnaire Development

A FDM questionnaire was developed based on literature review. Databases such as ScienceDirect, IEEE Explore, PubMed and Web of Science were utilized to search for related literatures on the identified variables which in this study were the four main roles, teacher, researcher, administrator and clinician's definitions. In this questionnaire, each of the variables is referred to as a major element or a construct and each of the statements related to the variables are referred to as a sub-element or an item under construct (Fig 1).

Figure 1: An example of the Major Element and Sub-elements in the FDM questionnaire

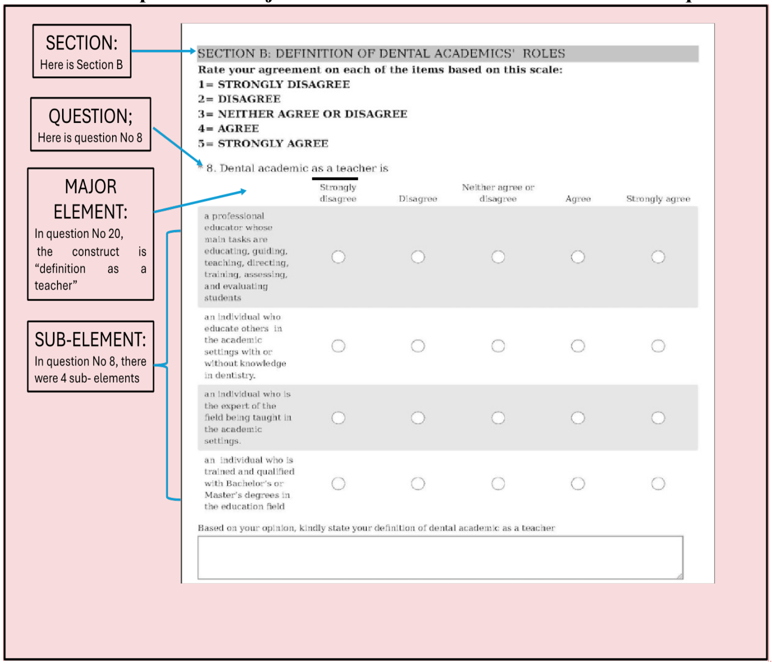

For an item to be incorporated within a construct, it must be supported by the existing literature. Consequently, a literature mapping defining the various roles of dental academics was devised (Table 2).

Table 2: Literature mapping for the definition of different dental academics' roles

MAJOR ELEMENT	SUB-ELEMENT	REFERENCES
Definition of a teacher	An educator whose main tasks are educating, guiding, teaching, directing, training, assessing, and evaluating students [21]	Saskianti et al.,2021
	An individual who educates others in the academic settings with or without knowledge in dentistry[22]	Radford et al., 2015
	An individual who is the expert of the field being taught in the academic settings [23]	Stenfors-Hayes et al, 2012
	An individual who is trained and qualified with Bachelor's or Master's degrees in the education field [24]	McConnell et al., 2021
Definition of a researcher	An individual with specific research skills and specific research-related attributes [25]	Laidlaw et al., 2012

Commented [sm7]: The image is not clear. Please insert a clear image. Not sure if it is my device. Please check to make sure image is clear

	An active member of the Research Centre and/or primary investigator in at least one research project with human participants [26]	Cumyn et al., 2019
	an individual who conducts research and provides direct patient care, although not necessarily at the same time or for the same organisation [27]	Hay-Smith et al., 2016
	An individual whose primary tasks are to teach and conduct research at the university level [28]	Carter, 2019
Definition of an administrator	An individual with knowledge, skills, and competencies regarding administration, managerial processes, legal and financial procedures related to an institution [29]	Fanoos & He, 2021
	An individual who is appointed as programme directors, course coordinators, curriculum directors or clinical coordinators [30]	Weng & Tang, 2014
	An individual who is expected to manage and lead the educational institution [24]	McConnell et al., 2021
	An individual who retains a clinical role while also engaging in management related activities, such as strategic and collaborative work with health care managers and professionals [31]	Edmonstone, 2005
Definition of a clinician	A healthcare professional that works in the healthcare setting [32]	Sherbino et al., 2014
	An individual who delivers safe, efficient patient care, whist supervising and teaching students and trainees [33]	Cleland et al., 2018
	An individual who is trained and highly skilled in clinical dentistry fields [34]	Kennedy and Hunt, 1999
	An individual who serves as a clinical teacher in the academic settings [33]	Cleland et al., 2018

The FDM questionnaire was designed using the SurveyMonkey platform (SurveyMonkey Inc., San Mateo, CA, USA; www.surveymonkey.com). It provided instructions for the expert panellists, detailing the purpose, duration, and objectives of the study, the investigator's contact information, and an overview of the Delphi process. The questionnaire was pre-tested and amendments made before piloted with 12 dental academics. The internal consistency of the items within the construct was evaluated using Cronbach's alpha (α), standard deviation (S), inter-item correlation (r), and corrected item-total correlation (CITC).

Commented [sm8]: Did the piloted questionnaire consider inclusion and exclusion criteria?

Commented [sm9]: Were the academics serving in different roles?
Were they from the same institution/ departments? Clarification required to determine if there is cross-cultural consensus

Commented [sm10]: The results of the pilot are not stated anywhere.

Expert Panellists Recruitment

Based on the inclusion and exclusion criteria (Table 3), invitations were sent to 37 experts from three countries based on their email address taken for their institutional website. Base on their

Commented [sm11]: How where these experts identified considering that results heavily relies on the expertise and diversity of the chosen panel,? Was it via professional network, are they in the same institution(s) as the authors, was it via literature review for credibility and reputation?I think just to say based on the email address is not enough.

Commented [sm12]: From the three countries how many institutions were incorporated?

consensus to participate, the FDM questionnaire was then distributed via the Survey Monkey platform.

Commented [sm13]: Was anonymity maintained if so how?

Table 3: Inclusion and exclusion criteria for the FDM rounds

Delphi panellist Inclusion Criteria	Delphi panellist Exclusion Criteria
Clinician and scientist who is currently serving or has previously served as an academic staff at a dental institution for more than 5 years.	Clinician and scientist who has not served as an academic staff at dental institution .
Lead administrative officer who is currently serving or has previously served as an administrative staff at dental institution for more than 5 years.	Clinician and scientist who is currently serving or has previously served as an academic staff at dental institution for less than 5 years.
A dental consultant who is currently serving or has previously served as a curriculum panellist at dental institution.	Lead administrative officer who is currently serving or has previously served as administrative staff at dental institution for less than 2 years.
Has experiences as a leader or coordinator of a programme.	Dental consultant/ dentist who has not served as a curriculum panellist at dental institution.
	Has no experience as leader or coordinator of any educational programme.

Commented [sm14]: This exclusion is not clear. Are we also to assume that clinicians in point 1 are/ have been a leader or coordinator?

Fuzzy Delphi Methods Data Analysis

All responses were compiled and tabulated in a Microsoft Excel worksheet and then transferred into the FDM Data Analysis template in Microsoft Excel (version 16.80). The first step in data analysis involved converting all linguistic variables into triangular fuzzy numbers (TFN). This process entails transforming the scales of the linguistic variables into TFN which were represented by the values m1, m2, and m3. Here, m1 corresponds to the minimum value, m2 to the most appropriate value, and m3 to the maximum value. In this study, the FDM questionnaire employed a 5-points Likert Scale and were converted into Fuzzy Scale based on their corresponding linguistic variable. [35,36,37]. Accordingly, each expert's response was mapped to its corresponding TFN within the Fuzzy Scale as presented in Table 4

Table 4: Conversion of Likert scale to Fuzzy Scale

Likert scale	Linguistic Variables	Fuzzy Scales		
1	Strongly Disagree	0.0	0.0	0.2
2	Disagree	0.0	0.2	0.4

3	Neither Agree nor Disagree	0.2	0.4	0.6
4	Agree	0.4	0.6	0.8
5	Strongly Agree	0.6	0.8	1.0

The Likert score given by each of the experts were inserted into the FDM analysis template and matched to their TFN and the average of m1, m2 and m3 were obtained for each of the items in the questionnaire. The difference between the experts' evaluation data and the average value for each item, identified as the threshold value, 'd' was calculated using the formula as below:

$$d(\tilde{m},\tilde{n}) = \sqrt{\frac{1}{3}[(m_1 - n_1)^2 + (m_2 - n_2)^2 + (m_3 - n_3)^2]}$$

Where :

d = threshold value

m1 = average of minimum value — n1 = minimum value
m2 = average of plausible value — n2 = plausible value
m3 = average of maximum value — n3 = maximum value

This threshold "d" value is important in determining the levels of agreement among the panel experts upon the items. Following the establishment of the threshold value, the expert agreement percentage was computed for each item. The determination of group consensus was calculated by using the formula below [38]:

$$\frac{\text{Total of } d \leq 0.2 \times 100}{n \text{ of experts}}$$

Commented [sm15]: Why is 0.2 chosen?

It was specified that the group consensus percentage should surpass 75% for the subsequent steps to proceed. Upon reaching a collective consensus within the group, the defuzzification process was carried out using an average of fuzzy numbers. This calculation aims to determine the value of the fuzzy score (A), as well as the rank and priority of each item based on this formula:

Commented [sm16]: I am not sure I get how the priority is determined with this formula. Please elaborate
Does it mean that the item with the highest fuzzy score is also considered to be priority?

$$A = \frac{1}{3}(m_1 + m_2 + m_3)$$

The item with the highest defuzzification or Fuzzy Score value (∝-cut value) will be ranked as the first item in the list. The fuzzy score value (A) must exceed the alpha cutting value of 0.5 (α-cut value > 0.5), for the item to be considered acceptable [39,40].

It is important to note that all three conditions for FDM analysis must be fulfilled for the items to be accepted. The three requirements are, the threshold d value must be ≤ 0.2, the group consensus percentage must be > 75%, and the fuzzy score (∝-cut value) should be > 0.5.

Delphi Rounds

The number of rounds was determined based on the FDM analysis and the group consensus percentage. Once consensus exceeded 75% and data saturation was reached, no additional rounds were required. The same protocol and coordination were applied throughout all FDM rounds.

Results

Fuzzy Delphi Method Questionnaire Reliability

Based on the reliability analysis, the major element "definition as a clinician" achieved an alpha (α) of 0.760, while "definition as a researcher" attained an alpha (α) of 0.775, both indicating acceptable reliability values. Similarly, "definition as an administrator" scored an alpha (α) of 0.806, and "definition as a teacher" scored an alpha (α) of 0.808, demonstrating a good level of reliability. All the items demonstrate reliability, supporting the retention of the pre-tested and validated set of questions for the Delphi rounds (Table 5). The finalized questionnaire comprised of 16 items representing various definitions of dental academics' roles.

Table 5: Internal consistency of the major elements in the FDM questionnaire

Constructs	Cronbach's alpha, □ (□ > 0.7)	Standard deviation, S (S>0)	Inter-item correlation, r (0.3>r<0.9)	Minimum Corrected Item-Total Correlation (CITC) (CITC >0.3)
Definition as a teacher (4 items)	0.808	0.45227– 0.51493	0.408 – 0.837	0.422
Definition as a researcher (4 items)	0.775	0.45227 – 0.51493	0.393- 0.683	0.446
Definition as an administrator (4 items)	0.806	0.49237 – 0.57735	0.426-0.746	0.510
Definition as a clinician (4 items)	0.760	0.49237 – 0.75378	0.490- 0.816	0.404

Expert Panellists Recruitment

The recruitment process for expert panellists yielded a favourable outcome, with approximately 73% of the invited experts (27 in total) agreeing to participate in the study. The initial panel consisted of 24 experienced dental academics, two senior clinical consultants affiliated with the Ministry of Health in Malaysia, and one senior administrator from a public institution. Following the first round, a second round of the Delphi method was conducted based on the analysis from the initial round. All 27 experts were invited to participate again via email; however, only 23 experts responded, resulting in an 85.2% response rate for this round. The composition of the second panel remained largely the same, with the exception of four experienced dental academics who withdrew.

Commented [sm17]: Are these academics who have been coordinators of a program as per inclusion and exclusion criteria?

Commented [sm18]: Was the administrator a dental academic? What does public institution mean? Is it teaching or treatment facility? Important to clarify considering that in other countries there are administrative staff who are not academics and are considered support staff with no dental training background.

First FDM Round Data Analysis

Based on the first FDM round (FDM 1), five out of the 16 response items did not comply with the FDM requirements (highlighted in Table 6). The FDM analysis revealed that item number 2, with a minimal 41% consensus among experts, a threshold value d=0.37, and a Fuzzy score (A) α=0.447, failed to meet the criteria of >75% expert consensus, d≤0.2, and α-cut value >

0.5. Similar findings were observed for item number 6. Regarding items number 4, 8, and 12, they did not meet two of the requirements. The three items scored d > 0.2 and group consensus < 75% (Table 6). Consequently, all five items were excluded from the list while the other 11 items were kept for the second FDM round.

Commented [sm19]: It will be interesting to know how this items fared in the pilot study? Especially Item 2 & 4 Items that will be highly influenced by practices in different countries and institutions? Which should be factored in.

Table 6: First FDM analysis with the excluded items highlighted

[redacted]	Triangular Fuzzy Nu nber		Defuzzification Process				[redacted]		
Ite ns	Threshold value, d	Group consensus , %	m1	m2	m3	Fuzzy Score (A)	Exper Consensu	∝-cut value	Ranking based on definition
1	0.10	96%	0.56	0.76	0.96	0.76296	ACCEPT	0.763	2
2	0.37	41%	0.27	0.44	0.64	0.44691	REJECT	0.447	16
3	0.15	96%	0.53	0.73	0.93	0.72593	ACCEPT	0.726	3
4	0.31	52%	0.31	0.50	0.70	0.50617	REJECT	0.506	14
5	0.18	85%	0.45	0.65	0.85	0.65185	ACCEPT	0.652	9
6	0.28	59%	0.28	0.47	0.67	0.47654	REJECT	0.477	15
7	0.13	89%	0.41	0.61	0.81	0.614815	ACCEPT	0.615	11
8	0.26	56%	0.36	0.56	0.76	0.562963	REJECT	0.563	13
9	0.17	93%	0.47	0.67	0.87	0.666667	ACCEPT	0.667	7
10	0.17	93%	0.48	0.68	0.88	0.681481	ACCEPT	0.681	6
11	0.18	93%	0.47	0.67	0.87	0.666667	ACCEPT	0.667	8
2	0.20	44%	0.40	0.60	0.80	0.600000	REJECT	0.600	12
13	0.20	81%	0.44	0.64	0.84	0.644444	ACCEPT	0.644	10
14	0.06	100%	0.58	0.78	0.98	0.777778	ACCEPT	0.778	1
15	0.18	93%	0.51	0.71	0.91	0.711111	ACCEPT	0.711	4
16	0.19	93%	0.50	0.70	0.90	0.703704	ACCEPT	0.704	5

A mapping system was devised to discern the items in the FDM questionnaire according to their respective numbers. This mapping proved valuable for scrutinizing the items in light of

the first FDM analysis and for the formulation of the second FDM questionnaire. The noncompliant items were highlighted in Table 7.

Table 7: Frst FDM analysis mapping with the excluded items highlighted

ROLES	ITEMS NUMBER	DEFINITION ITEMS	FDM RANKING
Teacher	1	An educator whose main tasks are educating, guiding, mentoring, teaching, directing, training, assessing, and evaluating students on the knowledge of dentistry and other related sciences	2
	2	A professional with a bachelors/master's qualification in dentistry and a mandatory basic training in teaching	16
	3	An individual who is the expert of the field being taught in the academic settings.	3
	4	An individual who is trained and qualified with Bachelor's or Master's degrees in the education field	14
Researcher	5	An individual with specific research skills and specific research-related attributes.	9
	6	An active member of the Research Centre and/or primary investigator in at least one research project with human participants	15
	7	An individual who conducts research and provides direct patient care, although not necessarily at the same time or for the same organisation	11
	8	An individual whose primary tasks are to teach and conduct research at the university level.	13
Administrator	9	An individual with knowledge, skills, and competencies regarding administration, managerial processes, legal and financial procedures related to an institution.	7
	10	An individual who is appointed as programme directors, course coordinators, curriculum directors or clinical coordinators	6
	11	An individual who is expected to manage and lead the educational institution.	8
	12	An individual who maintains a clinical role while engaging in management- related activities, such as strategic and collaborative work with managers and health care professionals.	12
Clinician	13	A healthcare professional that works in the healthcare setting	10
	14	An individual who delivers safe, efficient patient care, whilst supervising and teaching students and trainees	1
	15	An individual who serves as a clinical teacher in the academic settings.	4
	16	An individual who is trained and highly skilled in clinical dentistry fields.	5

Second FDM Round Data Analysis

In the second round of the Delphi method (FDM 2), both quantitative and qualitative findings from the initial round (FDM 1) were considered. The second FDM questionnaire was developed based on the 11 items approved in FDM 1, supplemented by an additional nine items generated from expert feedback. This resulted in a comprehensive questionnaire comprising a total of 20 items, with 23 experts remaining as panellists. The FDM analysis revealed that three items did not meet the established criteria for retention (see Table 8).

Commented [sm20]: How was the individual expert opinions aggregated and adjusted towards consensus?

Table 8: Second FDM analysis with the excluded items highlighted

	Triangular Fuzzy Number		Defuzzification Process						
Items	Threshold value, d	Group consensus , %	m1	m2	m3	Fuzzy Score (A)	Expert Consensus	∝-cut value	Ranking based on definition
1	0.09	100%	0.57	0.77	0.97	0.76522	ACCEPTED	0.77	1
2	0.13	100%	0.54	0.74	0.94	0.73913	ACCEPTED	0.74	4
3	0.20	78%	0.46	0.66	0.86	0.66087	ACCEPTED	0.66	10
4	0.31	39%	0.37	0.57	0.77	0.56522	REJECTED	0.57	18
5	0.20	87%	0.46	0.66	0.86	0.66087	ACCEPTED	0.66	10
6	0.18	96%	0.48	0.68	0.88	0.67826	ACCEPTED	0.68	8
7	0.17	96%	0.47	0.67	0.87	0.66957	ACCEPTED	0.67	9
8	0.20	83%	0.43	0.63	0.83	0.63478	ACCEPTED	0.63	13
9	0.18	65%	0.39	0.59	0.79	0.59130	REJECTED	0.59	16
10	0.20	83%	0.43	0.63	0.83	0.62899	ACCEPTED	0.63	14
11	0.15	91%	0.43	0.63	0.83	0.63478	ACCEPTED	0.63	12
12	0.15	100%	0.50	0.70	0.90	0.69565	ACCEPTED	0.70	6
13	0.17	91%	0.44	0.64	0.84	0.64348	ACCEPTED	0.64	11
14	0.16	87%	0.43	0.63	0.83	0.62609	ACCEPTED	0.63	15
15	0.15	91%	0.44	0.64	0.84	0.64348	ACCEPTED	0.64	11
16	0.20	91%	0.49	0.69	0.89	0.68696	ACCEPTED	0.69	7
17	0.10	100%	0.56	0.76	0.96	0.75652	ACCEPTED	0.76	2
18	0.17	91%	0.51	0.71	0.91	0.71304	ACCEPTED	0.71	5
19	0.13	100%	0.54	0.74	0.94	0.73913	ACCEPTED	0.74	3
20	0.26	39%	0.39	0.59	0.79	0.59130	REJECTED	0.59	17

Notably, item number 4 achieved only a 39% group consensus, with a threshold value of d = 0.31, which exceeds the acceptable limit. Similarly, item number 20 also recorded a 39% group consensus and a d value of 0.260, both of which were above the acceptable threshold, leading to their exclusion from further consideration. While item number 9 satisfied two of the FDM criteria, it fell short in terms of group consensus, achieving only 65%, which is below the acceptable value of 75%. Consequently, this item was also excluded from the final list. The item mapping process proved instrumental in identifying these items that did not meet retention criteria, as detailed in Table 9.

Table 9: Second FDM analysis mapping with the excluded items highlighted

Roles	Items number	Definition	FDM ranking
Teacher	1	An educator whose main tasks are educating, guiding, mentoring, teaching, directing, training, assessing and evaluating students in the knowledge of dentistry and other related fields.	1
	2	An individual who is the expert of the field being taught in the academic settings and practices evidence-based dentistry, and evidence-based education.	4
	3	An individual who is trained and qualified with minimum qualification of a master's degree in dentistry and/or another related field.	10
	4	An individual who is trained and qualified in their scope of teaching with reasonable academic training and have a general overview of dentistry.	18
	5	An individual who has adequate clinical experience and is able to guide the students using teaching methods that are updated and relevant.	10
Researcher	6	An individual with specific research skills and specific research-related attributes whose secondary tasks are to conduct, teach/guide research projects for the improvement of patients, society, organisation and betterment of mankind.	8
	7	An individual who conducts research and develop new techniques or new knowledge that has the potential to change future dental practices.	9
	8	An individual who has the ability and passion to conduct research in their area of interest and provides direct patient	13
		care, although not necessarily at the same time or for the same organisation.	
	9	An individual whose primary tasks are to teach and conduct research related to learning materials, to develop the sciences for educational purposes and not limited to the university or/and community level.	16

	10	An individual with basic research skills and knowledge such that they could provide supervision to the students.	14
Administrator	11	An individual with knowledge, leadership skills, and competencies regarding administration, managerial processes, legal and financial procedures related to an institution.	12
	12	An individual who may be appointed as programme directors, course coordinators, curriculum directors or clinical coordinators and helps to develop curriculum and manages academic and student programme.	6
	13	An individual who is expected to manage and lead the educational institution which involves strategic and collaborative activities.	11
	14	An individual who is appointed to carry out managerial or administrative work within the academic institution to facilitate the running and operations of academic, clinical, research and collaborative work so that other people (academicians/support staffs, students) can do their job well.	15
	15	An individual who is responsible for the dental program or parts of the program, talent management and overall operations of the dental school at the level suited for the appointment.	11
Clinician	16	A healthcare professional who works in the healthcare setting and involves directly in patient management, whether in clinic, hospital wards or operating theatres and actively provide treatment for patients to improve their wellbeing.	7
	17	An individual who serves as a clinical teacher in academic settings, and delivers safe, efficient patient care, whilst supervising and teaching students and trainees	2
	18	A specialist who is trained and highly skilled in clinical dentistry fields.	5
	19	An individual who is responsible in educating dental students, dental professionals, providing direct patient care and involved in its relationship with other disciplines which may be non-dental for the holistic care of patients.	3
	20	An individual who is trained in basic dental skills such that they can impart their skills to the students and deliver optimum health care standard themselves with passion for continuous education/ training.	17

In the second round of the Delphi method (FDM 2), consensus was achieved on 17 items among the 23 participating experts, encompassing the four primary roles of dental academics The absence of further input indicated that data saturation had been attained. The accepted items are systematically ranked and presented in Figure 2.

Commented [sm21]: There are items in bold what do those represent, please specify?

Figure 2: The definitions of different dental academic’s roles based on the FDM ranking

DEFINITION			
**TEACHER**	**RESEARCHER**	**ADMINISTRATOR**	**CLINICIAN**
an educator whose main tasks are **educating, guiding, mentoring, teaching, directing, training, assessing and evaluating students** in the knowledge of dentistry and other related fields.	an individual with **specific research skills and specific research-related attributes** whose **secondary tasks are to conduct, teach/guide research projects** for the improvement of patients, society, organization and betterment of mankind.	an individual who may be **appointed as programme directors, course coordinators, curriculum directors or clinical coordinators** and helps to **develop curriculum** and manages academic and student programme.	an individual who serves as **a clinical teacher** in academic settings, and **delivers safe, efficient patient care, whilst supervising** and teaching students and trainees
an individual who is **the expert of the field being taught** in the academic settings and practices evidence-based dentistry, and evidence-based education.	an individual who **conducts research and develop new techniques** or **new knowledge** that has the potential to change future dental practices.	an individual who is expected to **manage and lead the educational institution** which involves strategic and collaborative activities.	an individual who is **responsible in educating dental students, dental professionals,** providing direct patient care and involved in its relationship with other disciplines which may be non-dental for the holistic care of patients.
an individual who is **trained and qualified** with minimum qualification of a Master’s **degree in Dentistry and/or another related field.**	an individual who **has the ability and passion to conduct research** in their area of interest and provides direct patient care, although not necessarily at the same time or for the same organisation.	an individual who is **responsible for the dental program** or parts of the program, **talent management and overall operations of the dental school** at the level suited for the appointment.	a specialist who is **trained and highly skilled in clinical dentistry fields.**
an individual who has **adequate clinical experience** and is **able to guide the students** using teaching methods that are updated and relevant.	an individual with **basic research skills and knowledge** such that they **could provide supervision to the students**	an individual with **knowledge, leadership skills, and competencies** regarding **administration, managerial processes, legal and financial procedures** related to an institution.	**a healthcare professional** who **works in the healthcare setting and involves directly in patient management**, whether in clinic, hospital wards or operating theatres and actively provide treatment for patients to improve their wellbeing.
		An individual who is **appointed to carry out managerial or administrative work** within the academic institution to facilitate the running and operations of academic, clinical, research and collaborative work **so that other people (academicians/support staffs, students) can do their job well.**	

Discussion

As dental academics strive to balance the multifaceted nature of their roles, it creates a demanding work environment which may lead to academic identity crisis [41], low job satisfaction and impacted career progression. Additionally, the absence of clear career pathways [42] and limited literature defining faculty competencies and roles in medical and dental education [43] have contributed to a significant gap in the literature.

Commented [sm22]: In what way? May be once again dependant on country to country or institution to institution. A blanket approach might not suffice.

The study cited in my understanding was more on lack of awareness for about the career pathways in academia. Not sure if we are read it the same.

You can check out this article for some perspective of what's happening in other countries.

Mostert VC. Reasons why South African dentists chose a career in Dentistry, and later opted to enter an academic environment. S. Afr. dent. j. [Internet]. 2018 Apr [cited 2025 Jan 30] ; 73(3): 141-145. Available from: http://www.scielo.org.za/scielo.php?script=sci_arttext&pid=S0011-85162018000300006&lng=en.

In the attempt to understand and defined dental academics roles, the Fuzzy Delphi Method was employed. This approach was proposed by Murray et al. (1985) to enhance consensus-building among experts by incorporating fuzzy logic, which allows for more nuanced interpretations of their responses [44]. By using triangular fuzzy numbers, FDM enables a more precise representation of expert consensus, facilitating clearer decision-making and enhancing the reliability of the findings by improving the accuracy of the analysis [35]. This methodology is particularly valuable in contexts where expert opinions are critical, as it allows for a comprehensive understanding of complex issues while minimizing misinterpretations.

The careful selection of experts for the FDM rounds is crucial because the Delphi method does not require random statistical sampling; instead, participants must be chosen from qualified experts. While non-random sampling can introduce bias, the use of purposive sampling with strict inclusion and exclusion criteria in this study ensures sufficient diversity and transparency, mitigating this bias [45]. Furthermore, recruiting experts included clinicians and scientists with over 5 years of experience in academia, qualifies someone as an expert [46]. The heterogeneous panel of experts added in capturing a broad range of opinions. Involving experts and

stakeholders from different geographical regions and areas of expertise enhanced the scientific robustness of the findings [47,48].

Commented [sm23]: Which stake holders where involved, I might have missed it?

The definitions of dental academics, as derived from expert consensus, reveal a nuanced understanding of their roles and responsibilities within the educational landscape. The application of the FDM has illuminated the multifaceted nature of these roles, indicating that they cannot be encapsulated by a singular definition. Dental academics serve primarily as teachers, guiding and mentoring students while assessing their progress, all underpinned by their expertise in the field. In addition to their teaching responsibilities, they are also researchers, equipped with specialized skills that enable them to conduct research and foster innovation in dental techniques and knowledge. Furthermore, as administrators, dental academics take on leadership roles such as program directors and curriculum coordinators, where they are instrumental in shaping educational policies and managing institutional operations. Lastly, in their capacity as clinicians, they provide critical patient care while simultaneously educating the next generation of dental professionals. This comprehensive framework underscores the complexity of dental academia and highlights the importance of recognizing the diverse competencies required for effective practice in each role. Such insights are essential for developing targeted faculty development programs that enhance the professional identities and effectiveness of dental educators.

The findings from this study not only enhance our understanding of these roles but also underscore the importance of creating supportive institutional structures that promote career satisfaction and growth among dental faculty [49]. By clarifying these roles, dental schools can better tailor faculty development programs to address the diverse competencies required for success in academia.

Through these definitions, individual dental academic may also reflect who they are or who they have been. This is crucial for initiating career planning and managing career transitions [50,51]. It provides a foundational platform for dental academics to align their current or past identities with their future career goals [50]. In addition, these definitions provide a framework to help dental academics adapt to their multifaceted responsibilities and to support their professional advancement [43]. Furthermore, these findings may act as a self-reflective support tool to establish their academic identity, emphasize the need for dental institutions to invest in targeted faculty development programs that address the broad competencies required for success in academia [12].

Conclusion

A single definition was not discernible for each of the roles, given that each primary role encompassed a diverse range of job descriptions. Based on expert consensus, the following definitions outline the various roles within dental academia:

Dental academic as a teacher can be defined as an educator whose main tasks are educating, guiding, mentoring, teaching, directing, training, assessing and evaluating students in the knowledge of dentistry and other related fields or an individual who is the expert of the field being taught in the academic settings and practices evidence-based dentistry, and evidence based education.

Dental academic as a researcher can be defined as an individual with specific research skills and specific research-related attributes whose secondary tasks are to conduct, teach/guide

research projects for the improvement of patients, society, organization and betterment of mankind or an individual who conducts research and develop new techniques or new knowledge that has the potential to change future dental practices.

Dental academic as an administrator can be defined as an individual who may be appointed as programme directors, course coordinators, curriculum directors or clinical coordinators and helps to develop curriculum and manages academic and student programme or an individual who is expected to manage and lead the educational institution which involves strategic and collaborative activities.

Dental academic as a clinician can be defined as an individual who serves as a clinical teacher in academic settings, and delivers safe, efficient patient care, whilst supervising and teaching students and trainees or an individual who is responsible in educating dental students, dental professionals, providing direct patient care and involved in its relationship with other disciplines which may be non-dental for the holistic care of patients.

Acknowledgments

This study was self-funded by the first author as part of her doctoral research. We extend our gratitude to all the expert panellists for their invaluable support and contributions to this research.

Author Contributions

Conceptualisation, data curation and data analysis: Norasmatul Akma Ahmad

Methodology: Norasmatul Akma Ahmad &Zahra Naimie

Project administration: Norasmatul Akma Ahmad, Noor Hayaty Abu Kasim, Sabri Musa

Supervision: Noor Hayaty Abu Kasim, Sabri Musa, Siti Adibah Othman

Writing – original draft: Norasmatul Akma Ahmad
Writing – review & editing: Norasmatul Akma Ahmad, Noor Hayaty Abu Kasim, Sabri Musa, Siti Adibah Othman, Zahra Naimie

References

1. Pape G, Dong F, Horvath Z. Assessing the professional identity of dental school faculty: An exploratory study. J Dent Educ. 2018;82(11):1140-1145.
2. Wilkerson L, Irby DM. Strategies for improving teaching practices: A comprehensive approach to faculty development. Acad Med. 1998;73(4):387-396.
3. McLean M, Ashwin P. The quality of learning, teaching, and curriculum. New languages and landscapes of higher education. 2016:84-102.
4. van Dijk EE, Geertsema J, van der Schaaf MF, van Tartwijk J, Kluijtmans M. Connecting academics' disciplinary knowledge to their professional development as university teachers: A conceptual analysis of teacher expertise and teacher knowledge. High Educ. 2023;86(4):969-984.
5. Chuenjitwongsa S, Oliver RG, Bullock AD. Competence, competency-based education, and undergraduate dental education: A discussion paper. Eur J Dent Educ. 2018;22(1):1-8.
6. Hoskin ER, Bertone M, Chun YHP, Lee AL, Motahari MZ, Martin AB. Should dental school faculty be measured and compensated using academic productivity models? Two viewpoints. J Dent Educ. 2020;84(5):534-542.

7. Haden NK, Hendricson WD, Killip JW, O'Neill PN, Reed MJ, Weinstein G, et al. Developing dental faculty for the future: ADEA/AAL institute for teaching and learning, 2006–09.
8. Scott J. Dental education in Europe: The challenges of variety. J Dent Educ. 2003;67(1):69-78.
9. Hand JS. Identification of competencies for effective dental faculty. J Dent Educ. 2006;70(9):937-947.
10. Dugas D, Stich AE, Harris LN, Summers KH. 'I'm being pulled in too many different directions': Academic identity tensions at regional public universities in challenging economic times. Stud High Educ. 2020;45(2):312-326.
11. Piano M, Diemer K, Hall M, Hui F, Kefalianos E, Lawford BJ, et al. A rapid review of challenges and opportunities related to diversity and inclusion as experienced by early and mid-career academics in the medicine, dentistry and health sciences fields. BMC Med Educ. 2023;23(1):288.
12. Sabato E, Doubleday AF, Lee CT, Correa LP, Huja S, Crain G. Recommendations for remaining agile in the face of a dental faculty workforce shortage. J Dent Educ. 2023;87(3):295-302.
13. Wei LZ, Zhou SS, Hu S, Zhou Z, Chen J. Influences of nursing students' career planning, internship experience, and other factors on professional identity. Nurse Educ Today. 2021;99:104781.
14. Herman N, Jose M, Katiya M, Kemp M, Le Roux N, Swart-Jansen van Vuuren C, Van der Merwe C. 'Entering the world of academia is like starting a new life': A trio of reflections from health professionals joining academia as second career academics. Int J Acad Dev. 2021;26(1):69-81.

15. Stone S, Ellers B, Holmes D, Orgren R, Qualters D, Thompson J. Identifying oneself as a teacher: The perceptions of preceptors. Med Educ. 2002;36(2):180-185.
16. Mao J, Shen Y. Identity as career capital: Enhancing employability in the creative industries and beyond. Career Dev Int. 2020;25(2):186-203.
17. Lieff S, Baker L, Mori B, Egan-Lee E, Chin K, Reeves S. Who am I? Key influences on the formation of academic identity within a faculty development program. Med Teach. 2012;34(3):e208-e215.
18. Spurk D, Hirschi A, Dries N. Antecedents and outcomes of objective versus subjective career success: Competing perspectives and future directions. J Manag. 2019;45(1):35-69.
19. Saffie NAM, Rasmani KA. Fuzzy Delphi method: Issues and challenges [Internet]. In: Proceedings of the 2016 International Conference on Logistics Informatics and Service Sciences (LISS); July 2016; pp 1-7.
20. Yusof N, Hashim NLH, Hussain A.A review of fuzzy Delphi method application in human-computer interaction studies [Internet]. In: AIP Conference Proceedings; August 2022; Vol 2472 No 1.
21. Saskianti T et al.. Teachers' role in regular and special need students' oral health: A narrative review. J Int Dent Med Res..2021;14(4):1639-1643.
22. Radford DR et al.. Identifying and preparing the next generation of part-time clinical teachers from dental practice. Brit Dent J..2015;219(7):319-322.
23. Stenfors-Hayes T et al.. Three ways of understanding development as a teacher. Eur J Dent Educ..2012;16(1):e151-e157.
24. McConnell III JR et al.. Preservice Teachers' Performance and Confidence in Their Readiness to Teach: An Exploratory Study. SRATE Journal..2021;30(1):n1.

25. Laidlaw A et al.. Developing research skills in medical students: AMEE Guide No 69.Med Teach..2012;34(9):754-771.

26. Cumyn A et al.. Role of researchers in the ethical conduct of research: A discourse analysis from different stakeholder perspectives. Ethics Behav..2019;29(8):621-636.

27. Hay-Smith EJC et al.. Once a clinician always a clinician: A systematic review to develop a typology of clinician-researcher dual-role experiences in health research with patient-participants.BMC Med Res Methodol..2016;16:1-17.

28. Carter SD.Increased workforce diversity by race gender and age and equal employment opportunity laws: Implications for human resource development. Gender Divers Concepts Methodol Tools Appl..2019;380-405.

29. Fanoos A He Y. Curriculum analysis of educational leadership master's programs in the university system of Maryland. Educ Manag Adm Leadership..2021;49(5):841-858.

30. Weng CH Tang Y. The relationship between technology leadership strategies and effectiveness of school administration: An empirical study. Computers Educ..2014;76:91-107.

31. Edmonstone J.Clincial Leadership Development.eBook Partnership.;2005.

32. Sherbino J Frank JR Snell L.Defining the key roles and competencies of the clinician–educator of the 21st century: A national mixed-methods study.Acad Med..2014;89(5):783-789.

33. Cleland J et al.. Using paradox theory to understand responses to tensions between service and training in general surgery.Med Educ..2018;52(3):288-301.

34. Kennedy JE Hunt RJ.Meeting the demand for future dental faculty.In:75th Anniversary Summit Conference Proceedings.Washington DC: American Association of Dental Schools.;1999.

35. Abdullah MRTL.Development of activity-based mLearning implementation model for undergraduate English language learning [Doctoral dissertation].University of Malaya.;2014.

36. Jailani MA.The Application of Fuzzy Delphi Method in Content Validity Analysis [No 4535].EasyChair.;2020.

37. Joudar SS Albahri AS Hamid RA.Intelligent triage method for early diagnosis autism spectrum disorder (ASD) based on integrated fuzzy multi-criteria decision-making methods.Informatics Med Unlocked..2023;36:101131.

38. Bui TD Tsai FM Tseng ML et al..Identifying sustainable solid waste management barriers in practice using the fuzzy Delphi method.Resources Conserv Recycl..2020;154:104625.

39. Mustapha R Darussalam G.Aplikasi kaedah fuzzy delphi dalam penyelidikan sains sosial.Penerbit Universiti Malaya.;2018.

40. Ab Kadir K Sahari@Ashaari N Ramli RZ Salim J.Islamic information credibility scale development: Factors and indicators validation using fuzzy Delphi technique.Inform Dev..2023;02666669231163255.

41. Dugas D Summers KH Harris LN Stich AE.Shrinking budgets growing demands: Neoliberalism and academic identity tension at regional public universities.AERA Open..2018;4(1):2332858418757736.

42. Hayes MJ Ingram K. Australian dental practitioner perspectives on academic careers.J Dent Educ..2021;85(3):341-348.

43. Krupp MM Barlow PB Kyle EJ.Developing a self-assessment tool for dental faculty to map professional growth.J Dent Educ..2021;85(10):1596-1605.

44. Murray TJ Pipino LL Van Gigch JP.A pilot study of fuzzy set modification of Delphi.Human Syst Manag..1985;5(1):76-80.
45. Devaney L Henchion M.Who is a Delphi ‘expert’? Reflections on a bioeconomy expert selection procedure from Ireland.Futures..2018;99:45-55.
46. Berliner DC.Expert teachers: Their characteristics development and accomplishments.Bull Sci Technol Soc..2004;24(3):200-212.
47. Hussler C Muller P Rondé P.Is diversity in Delphi panelist groups useful? Evidence from a French forecasting exercise on the future of nuclear energy.Technol Forecast Soc Change..2011;78(9):1642-1653.
48. Freitas Â Santana P Oliveira MD Almendra R Bana e Costa JC Bana e Costa CA.indicators for evaluating European population health: A Delphi selection process.BMC Public Health..2018;18:1-20.
49. Sharab L Sonkar J Thomas PM Prasannakumar P Guha U Leventer M et al.Reshaping dental faculty development using collective healthcare experiences.J Dent Educ..2023;87(9):1234-1241.
50. Del Corso J Rehfuss MC.The role of narrative in career construction theory.J Vocat Behav..2011;79(2):334-339.
51. Modestino AS Sugiyama K Ladge J.Careers in construction: An examination of the career narratives of young professionals and their emerging career self-concepts.J Vocat Behav..2019;115:103306.
