# Supplementary material for: Defining the multifaceted roles of dental academics: A consensus approach using the Fuzzy Delphi Method
Source: PLoS One. 2025 Aug 22;20(8):e0330636. doi: 10.1371/journal.pone.0330636 (PMC12373288; doi:10.1371/journal.pone.0330636)
Supplement: Attachment — Submitted filename: Response to reviewers- rev01.docx [file pone.0330636.s003.docx]

**Defining the Multifaceted Roles of Dental Academics: A Consensus Approach Using the Fuzzy Delphi Method**

| Reviewers’ Comments | Author’s Response |
| --- | --- |
| **Reviewer 1:**  This research addresses issues pertinent to dental academics in as far as their roles are concerned. Although the current literature was cited, it does not seem to reflect the global context. Some articles e.g. Ref 42 interpretation may need to be revised.  The study 42 cited with reference to career pathways in my understanding was more on lack of awareness about the career pathways in academia. Not sure the interpretation is correct.  You can check out this article for some perspective of what’s happening in other countries.  Mostert VC. Reasons why South African dentists chose a career in Dentistry, and later opted to enter an academic environment. S. Afr. dent. j. [Internet]. 2018 Apr [cited 2025 Jan 30] ; 73( 3 ): 141-145. Available from: http://www.scielo.org.za/scielo.php?script=sci_arttext&pid=S0011-85162018000300006&lng=en | Thank you for your comment.  We appreciate your observation regarding our interpretation of Reference 42. Upon review, we agree that the Hayes and Ingram study primarily highlights the lack of awareness and clarity regarding academic career pathways among dental clinicians in Australia. The study involved 85 dental clinicians, of whom 79% reported that there was no clear pathway to an academic career and this lack of a clear career pathway was perceived as a barrier to academic career. We have revised our manuscript to more accurately reflect this focus on perceived barriers and limited awareness and we have also included Mostert (2018) as a valuable comparative perspective.  The new paragraph with updated citations (page 23):  “Dental academics are required to manage a wide range of responsibilities including teaching, research, clinical duties, and administration which contributes to a demanding work environment. This complexity can lead to challenges such as academic identity crisis [42], reduced job satisfaction, and hindered career progression. Regardless of the factors that motivate clinicians to pursue academic careers, whether it is limited awareness and unclear pathways that may discourage entry [43], or the appeal of intellectual engagement and job security that may encourage it [44], defining the core roles of dental academics remains essential. Clear role definitions are important to support academic identity development, guide career progression, and inform institutional policies across diverse educational settings. Furthermore, the limited literature outlining faculty competencies and role expectations in medical and dental education [45] highlights a significant gap that this study seeks to address.” |
| The study design is appropriate, and has academic merit as it attempts to address an important topic that could address issues in dental education requiring some extent of committed and support to dental academics among key decision makers in dental education. | We thank the reviewer for recognising the academic merit of our study and its relevance to current challenges in dental education. We agree that addressing the multifaceted roles of dental academics is vital for informing institutional planning, resource allocation, and professional development strategies. It is our hope that the findings from this study will support efforts to guide decision-makers in better understanding the role-specific needs of dental academics and the importance of structured support systems for their career progression. |
| Details of methods and analysis applied in this study can be improved. It is not clear how the experts panellists where identified? | We appreciate this comment. We have revised the manuscript to more clearly describe how experts were purposively selected based on specific inclusion criteria, including a minimum of five years of academic experience and active involvement in multiple dental academic roles. To preserve participant anonymity, we have not listed individual names but have included a summary table outlining their country of origin, professional roles, and years of experience. This additional detail is now presented in the revised Methods section and in Table 4.  The new updated paragraph (page 9) :  “Based on the inclusion and exclusion criteria (Table 3), a purposive sampling strategy was used to identify potential experts from three countries. Expert panellists were identified through institutional websites and publicly available curricula vitae, with selection based on their active involvement in multiple academic roles such as teaching, research, clinical practice, and administration  Invitations were sent via email to 37 identified experts from 13 dental institutions and healthcare facilities, and those who consented to participate were subsequently provided with the Fuzzy Delphi Method (FDM) questionnaires through the SurveyMonkey platform. A summary of the demographic and professional characteristics of the expert panel is presented in Table 4.” |
| The results of the pilot study for the Fuzzy Delphi Method was not analysed and published. This results should have been published or presented as it would have provided valuable insights into the effectiveness of the questionnaire and justification for refining before conducting first round of FDM. This could also assist those wishing to repeat similar study. Result of the pilot study could contribute to the understanding of FDM application and potential challenges. | We appreciate the reviewer’s comment regarding the importance of presenting the pilot study findings. We would like to clarify that the results of the pilot study have been included in the manuscript under the "Fuzzy Delphi Method Questionnaire Reliability" subheading within the Results section.  In this section, we presented the reliability analysis of the initial questionnaire using Cronbach’s alpha, which demonstrated a high level of internal consistency. Additionally, we reported on how feedback from the pilot participants was used to refine the wording and structure of several items before launching the first round of the Fuzzy Delphi Method. This process ensured content clarity and improved instrument validity, which we agree is crucial for those seeking to replicate similar studies.  Nonetheless, based on the reviewer’s suggestion, we have reviewed this section and have made minor revisions to enhance its visibility and clarify the linkage between the pilot testing and subsequent questionnaire refinement.  In the updated method section (page 9) :  “The questionnaire was pre-tested and subsequently piloted with 12 dental academics who were not part of the main expert panel. This pilot study was conducted prior to the first round of the Fuzzy Delphi Method to assess the reliability, clarity, and relevance of the questionnaire items. Amendments were made based on pre-test feedback to improve wording and structure. During the pilot phase, the internal consistency of the constructs was evaluated using Cronbach’s alpha (α), standard deviation (SD), inter-item correlation (r), and corrected item-total correlation (CITC). Findings from the pilot test were used to refine item phrasing and ensure reliability before conducting the first round of the FDM. These results are reported under the 'Fuzzy Delphi Method Questionnaire Reliability' subheading.  In the updated results section (page 14) :  “Based on the reliability analysis conducted during the pilot study, all four main constructs demonstrated acceptable to good internal consistency. The major element “definition as a clinician” achieved an alpha (α) of 0.760, while “definition as a researcher” attained an alpha (α) of 0.775, both indicating acceptable reliability values. Similarly, “definition as an administrator” scored an alpha (α) of 0.806, and “definition as a teacher” scored an alpha (α) of 0.808, demonstrating a good level of reliability (Table 6). Given these findings, the questionnaire was deemed reliable and was retained without major modifications for use in the subsequent Delphi rounds. The finalised questionnaire consisted of 16 items representing various role definitions of dental academics. |
| There are no clearly defined objectives; the Fuzzy Delphi method is designed to gather expert consensus on a specific topic, so the research needs to have a clear goal in mind to guide the selection of experts, the questions asked, and the interpretation of results. These objectives should be aligned with the current knowledge and concerns within the field of study. | Thank you for your insightful comment. We have revised the manuscript to clearly state that this study aims to systematically define the specific roles of dental academics using the Fuzzy Delphi Method (FDM). This represents the first part of a larger research project that investigates the attributes and competencies required for each of the identified dental academic roles. By establishing role definitions as a foundation, this study supports subsequent phases that will explore the skills and competencies necessary for career development and professional growth in dental academia.  The new updated paragraph (pages 4-5) :  “This study represents the first phase of a larger research project aimed at developing a comprehensive Dental Academic Career Pathway (DACP) model. It seeks to systematically define the specific roles of dental academics using the Fuzzy Delphi Method (FDM), forming a foundational step toward understanding the attributes, competencies, and developmental needs within dental academia. Through expert consensus, this study offers essential role definitions that can support academic identity formation and inform institutional policy and professional development. While the questionnaire was constructed through literature mapping and expert input, the broader study adopts the Social Cognitive Career Theory (SCCT) as its conceptual lens. SCCT will be applied in later phases to interpret how clearly defined academic roles may influence self-efficacy, outcome expectations, and motivation to pursue or sustain a career in dental academia.” |
| There are a number of issues with the methods and analysis that need to be clarified/addressed; furthermore, some of the conclusions overreach the data collected, while other important results are given less emphasis that they warrant. How where the experts identified, and how was their annonimity maintained. It is not clear how the individual expert opinions were aggregated and adjusted towards a consensus. This should be reported. | Thank you for your feedback. We would like to clarify that this study employed the Fuzzy Delphi Method (FDM), in which each expert panellist was contacted individually and provided their responses independently via the Survey Monkey platform. There was no interaction or discussion among the experts, ensuring anonymity and reducing the risk of groupthink or dominant influence.  Consensus was not reached through group discussion but was instead determined quantitatively using the established FDM criteria. Specifically, an item was accepted only when all three of the following conditions were fulfilled:   1. The threshold (d) value was ≤ 0.2, 2. The group consensus percentage exceeded 75%, and 3. The fuzzy score (α-cut value) was > 0.5.   This rigorous process ensured objectivity and consistency in determining consensus. We have revised the relevant sections of the manuscript to more clearly explain these methodological details.  The new updated paragraph (page 13) :  “The consensus process in this study was conducted entirely through a quantitative approach. Each item in the questionnaire was analysed using the Fuzzy Delphi Method (FDM), and items were retained only if they met all three standard conditions: (1) the threshold value (d) reflecting the distance between experts’ opinions was ≤ 0.2; (2) the percentage of agreement among panellists exceeded 75%; and (3) the fuzzy score (α-cut value) was greater than 0.5. These thresholds are consistent with established FDM practices and were applied to ensure that only items with strong consensus and perceived relevance were included. This rigorous and structured approach reduces subjectivity and enhances the reliability and validity of the findings.” |
| It is overall a valuable study, in institutions where the different roles are not clearly defined. | We sincerely thank the reviewer for recognising the value of this study. Indeed, this was a key motivation behind our work, to systematically define the multifaceted roles of dental academics and address the ambiguity often observed in academic settings. By establishing clearer role definitions, we hope to support faculty development, improve institutional clarity, and contribute to the broader discourse on academic identity and progression in dental education. |
| **Reviewer 2:**  Paper is well written and structure. My quires and suggestions are given as follows: 1) What exact criteria or decision rules were used in selecting and modifying the items between FDM rounds? Please provide examples of how feedback was used to generate the 9 new items in Round 2. | We appreciate the reviewer’s request for greater clarity on the decision rules used between FDM rounds.  In the first round of the Fuzzy Delphi Method (FDM), each item was quantitatively evaluated against three core criteria:   1. The threshold value (d) ≤ 0.2 2. Expert consensus > 75% 3. Fuzzy score (α-cut value) > 0.5   Items failing to meet one or more of these criteria were excluded from Round 2.  In addition to the statistical analysis, qualitative feedback from expert panellists was critically reviewed. Experts were encouraged to provide open-ended comments, suggestions, and critiques on each role definition. This qualitative input was thematically analysed and used to guide refinement of existing items and development of new items.  For example, under the role of “Teacher,” Round 1 included the following four items:   1. An educator whose main tasks are educating, guiding, mentoring, teaching, directing, training, assessing, and evaluating students on the knowledge of dentistry and other related sciences. 2. A professional with a bachelor's/master’s qualification in dentistry and a mandatory basic training in teaching. 3. An individual who is the expert of the field being taught in the academic settings. 4. An individual who is trained and qualified with Bachelor’s or Master’s degrees in the education field.   Experts commented that these items did not sufficiently reflect clinical expertise, pedagogical training, or evidence-based teaching practices. In response, these items were revised for clarity and relevance, and new items were added. For instance:   - **Item 2** was refined to: *"An individual who is the expert of the field being taught in the academic settings and practices evidence-based dentistry, and evidence-based education."* - **Item 5 (new addition)** was created based on expert input emphasising the need for practical clinical teaching: *"An individual who has adequate clinical experience and is able to guide the students using teaching methods that are updated and relevant."*   These modifications reflect both the statistical outcomes and the expert recommendations and were intended to increase precision, relevance, and comprehensiveness of the constructs. |
| 2) Can the authors clarify how subjectivity in assigning fuzzy numbers was minimized? Was there any calibration or standardization step among raters? | Thank you for the valuable question. In this study, subjectivity in assigning fuzzy numbers was minimised by using a standardised and literature-supported conversion mapping between Likert scale responses and Triangular Fuzzy Numbers (TFNs). Experts were only required to respond using a 5-point Likert scale, and they did not assign any fuzzy values themselves, which removed individual variability in fuzzy interpretation. The conversion used was presented in Table 5: Likert scale \| Linguistic Variables \| Fuzzy Scales \| \|; 1 \| Strongly Disagree \| 0.0 \| 0.0 \| 0.2; 2 \| Disagree \| 0.0 \| 0.2 \| 0.4; 3 \| Neither Agree nor Disagree \| 0.2 \| 0.4 \| 0.6; 4 \| Agree \| 0.4 \| 0.6 \| 0.8; 5 \| Strongly Agree \| 0.6 \| 0.8 \| 1.0 This TFN structure was based on prior applications of the Fuzzy Delphi Method, particularly in educational and content validation studies [36–38]. By predefining these fuzzy values and applying them uniformly during analysis, we ensured consistency and minimised subjectivity in the quantification of expert consensus. As the fuzzy numbers were derived from fixed, validated scales, no further calibration among raters was necessary. |
| 3) More information is needed on the pre-testing phase of the FDM questionnaire (e.g., how many items were revised or removed, types of issues identified, specific actions taken). | Thank you for your comment. Based on your suggestion, we have reviewed this section and have made minor revisions to enhance its visibility and clarify the linkage between the pilot testing and subsequent questionnaire refinement.  In the updated method section (page 9):  “The questionnaire was pre-tested and subsequently piloted with 12 dental academics who were not part of the main expert panel. This pilot study was conducted prior to the first round of the Fuzzy Delphi Method to assess the reliability, clarity, and relevance of the questionnaire items. Amendments were made based on pre-test feedback to improve wording and structure. During the pilot phase, the internal consistency of the constructs was evaluated using Cronbach’s alpha (α), standard deviation (SD), inter-item correlation (r), and corrected item-total correlation (CITC). Findings from the pilot test were used to refine item phrasing and ensure reliability before conducting the first round of the FDM. These results are reported under the 'Fuzzy Delphi Method Questionnaire Reliability' subheading.  In the updated results section (page 14) :  “Based on the reliability analysis conducted during the pilot study, all four main constructs demonstrated acceptable to good internal consistency. The major element “definition as a clinician” achieved an alpha (α) of 0.760, while “definition as a researcher” attained an alpha (α) of 0.775, both indicating acceptable reliability values. Similarly, “definition as an administrator” scored an alpha (α) of 0.806, and “definition as a teacher” scored an alpha (α) of 0.808, demonstrating a good level of reliability (Table 6). Given these findings, the questionnaire was deemed reliable and was retained without major modifications for use in the subsequent Delphi rounds. The finalised questionnaire consisted of 16 items representing various role definitions of dental academics. |
| 4) While Table 2 maps references to role definitions, not all added or retained items in Round 2 seem clearly traceable to literature. Could the authors provide supporting references for each final item? | Thank you for your comment. We appreciate the opportunity to clarify the origin and development of the final items.  Table 2 in the manuscript represents the initial literature mapping, from which the preliminary items were derived. These items were grounded in previously published studies that explored the roles and functions of dental academics.  However, the final items retained after Round 2 of the Fuzzy Delphi Method (FDM) reflect the outcome of expert consensus, developed through a systematic and iterative validation process.  While not every final item can be directly traced to a specific reference in the literature, this is a key feature of the FDM as it facilitates the generation of new insights and refined constructs based on collective expert input.  The newly added or modified items, particularly those contributed through open-ended feedback from the expert panellists represent emergent knowledge generated through this rigorous methodology.  These validated items now serve as a novel contribution to the body of knowledge on defining the roles of dental academics. |
| 5) Can the authors position their findings more clearly within established academic/professional identity frameworks? For instance, how do their role definitions align with or differ from Boyer's model or Wenger's Communities of Practice? | We appreciate the reviewer’s thoughtful suggestion to position our findings within established academic/professional identity frameworks such as Boyer’s model or Wenger’s Communities of Practice.  While these frameworks offer valuable insights into academic identity and practice, this study This study is grounded based on the social cognitive career theory (SCCT) lens which was developed by Robert W. Lent, Steven D. Brown, and Gail Hackett in 1994. We adopted the SCCT as its primary conceptual lens as it provides a strong foundation for examining how personal factors (e.g., self-efficacy), contextual influences, and outcome expectations shape career development in academic settings.  Our focus was on defining the multifaceted roles of dental academics to support career clarity and informed career decision-making, an approach that aligns closely with the career progression and development aspects emphasised in SCCT.  Nevertheless, we acknowledge that the role definitions derived in this study may also contribute to the broader discourse on academic identity and could be further examined in relation to frameworks like Boyer’s and Wenger’s in future studies.  We have added a clarification in the introduction section to reflect SCCT as the conceptual lens.  The new updated paragraph (pages 4-5) :  “This study represents the first phase of a larger research project aimed at developing a comprehensive Dental Academic Career Pathway (DACP) model. It seeks to systematically define the specific roles of dental academics using the Fuzzy Delphi Method (FDM), forming a foundational step toward understanding the attributes, competencies, and developmental needs within dental academia. Through expert consensus, this study offers essential role definitions that can support academic identity formation and inform institutional policy and professional development. While the questionnaire was constructed through literature mapping and expert input, the broader study adopts the Social Cognitive Career Theory (SCCT) as its conceptual lens. SCCT will be applied in later phases to interpret how clearly defined academic roles may influence self-efficacy, outcome expectations, and motivation to pursue or sustain a career in dental academia.” |
| 6) The manuscript asserts that roles are distinct, yet many items blend responsibilities (e.g., clinician and teacher). Could the authors more clearly distinguish or justify this overlap in terms of role theory? | We thank you for the comment. While our study aims to define distinct roles within dental academia, we acknowledge that many responsibilities inherently overlap . For example, the clinician’s involvement in student clinical supervision blurs the line between clinical service and teaching.  This overlap is consistent with findings by Chuenjitwongsa et al. (2018), who highlighted that the roles of dental academics are interrelated and dynamic, often performed concurrently rather than in isolation. This interdependence reflects the realities of academic dental practice, where effective teaching often occurs within clinical settings, and research may inform both teaching and clinical care.  From a role theory perspective, particularly Katz and Kahn’s (1978) framework, this blending is not contradictory. Role sets in professional environments like academia are inherently fluid, and individuals may simultaneously fulfil multiple, overlapping roles depending on institutional context and expectations.  Accordingly, the blending observed in some item definitions (e.g., clinician and teacher) is deliberate, capturing the integrated nature of academic work rather than artificially isolating functions that are inseparable in practice.  In addition, both ‘Role Theory’ and ‘Social Cognitive Career Theory (SCCT)’ share a focus on how individuals internalise, perform, and develop within professional roles, making them complementary frameworks for understanding how dental academics form career identities and make progression decisions based on role clarity, expectations, and self-efficacy. |
| 7) The conclusion mentions support for academic identity and career planning. Could the authors provide a conceptual model or visual framework showing how these roles integrate into dental academic career stages? | Thank you for this valuable suggestion. We agree that a conceptual model illustrating how these defined roles integrate into academic career stages would be beneficial. However, as outlined in the introduction, this study represents the first phase of a larger research project aimed at developing a comprehensive Dental Academic Career Pathway (DACP)model.  In this initial phase, we focus solely on systematically defining the core roles of dental academics through expert consensus using the Fuzzy Delphi Method (FDM).  The integration of these role definitions into academic career stages along with their influence on attributes, competencies, and progression pathways will be presented in another manuscript as the final output of the larger study. |
| 8) Figure 2 is referenced but not adequately labeled or described. Can the authors ensure all figures and tables are self-explanatory and consistently formatted? | We appreciate the suggestion to enhance clarity and consistency in figure presentation. Figure 2 is currently titled "Figure 2: The definitions of different dental academic roles based on the FDM ranking and presented in table" and is intended to visually summarise the final role definitions established through the Fuzzy Delphi Method.  To improve clarity and ensure it is fully self-explanatory, we have revised the figure caption to more explicitly describe its content (page 22) :  “Figure 2: Final accepted definitions of dental academic roles. This figure presents the systematically ranked and categorised definitions of the four main roles as teacher, researcher, administrator, and clinician based on expert consensus using the Fuzzy Delphi Method.”  and added a brief explanatory note within the manuscript text when introducing the figure.  The new updated paragraph (pages 20-21) :  “The accepted items are systematically ranked and presented in Figure 2, which tabulates the final definitions for each of the four main dental academic roles, as teacher, researcher, administrator, and clinician as derived through the FDM consensus process.”  We have also reviewed all figures and tables to ensure consistent formatting and clearer labelling throughout the manuscript. |
| 9) Several passages could benefit from grammatical tightening and clarity (e.g., “The strive to balance” → “The effort to balance”; “has experiences” → “has experience”). Consider a language polish throughout. | Thank you for highlighting the need for improved grammatical clarity. We have carefully reviewed the manuscript and revised several passages for grammatical accuracy and language clarity, including correcting phrases such as “The strive to balance” to “The effort to balance” (page 2) and “has experiences” to “has experience” (page 10). A thorough language polish has been conducted throughout the manuscript to enhance overall readability and precision. |
